# Influence of axial limb rotation on radiographic lower limb alignment: a systematic review

**DOI:** 10.1007/s00402-021-04163-w

**Published:** 2021-10-01

**Authors:** Marc-Daniel Ahrend, Heiko Baumgartner, Christoph Ihle, Tina Histing, Steffen Schröter, Felix Finger

**Affiliations:** 1 Department of Traumatology and Reconstructive Surgery, BG Trauma Center Tübingen, Eberhard-Karls University of Tübingen, Schnarrenberg-Str. 95, 72076 Tübingen, Germany; 2 AO Research Institute Davos, Davos, Switzerland; 3 Department of Traumatology and Reconstructive Surgery, Diakonie Klinikum GmbH Jung-Stilling-Krankenhaus, Siegen, Germany

**Keywords:** Rotation, Limb alignment, Long-leg radiographs, Lower limb

## Abstract

**Introduction:**

The influence of limb malrotation on long-leg radiographs (LLR) is frequently discussed in literature. This systematic review aimed to describe the influence of limb rotation on alignment measurements alone and in combination with knee flexion, and determine its clinical impact.

**Materials and methods:**

A literature search was conducted in June 2021 using the databases MEDLINE, Cochrane, Web of Science (Clarivate Analytics), and Embase. The search term ((radiograph OR X-ray) AND (position OR rotation) AND limb alignment) was used. Database query, record screening, and study inclusion and exclusion were performed by two reviewers independently. Experimental studies (using either specimens or synthetic bones) or clinical studies (prospective or retrospective using radiographs of patients) analyzing the influence of limb rotation on anatomic and mechanical limb alignment measurements were included. Characteristics and results of the included studies were summarized, simplified, and grouped for comparison to answer the research question. Studies were compared descriptively, and no meta-analysis was performed.

**Results:**

A total of 22 studies were included showing large heterogeneity, comprising studies with cadavers, patients, and synthetic bones. Most studies (7 out of 8) reported that external rotation (ER) causes less apparent valgus and leads to more varus and internal rotation (IR) causes more valgus and leads to less varus. However, there is no consensus on the extent of rotation influencing alignment measures. Studies reported about an average change of > 2° (*n* = 4) and < 2° (*n* = 4) hip-knee-ankle angle (HKA) between 15°IR and 15°ER. There is a consensus that the impact of rotation on mechanical alignment is higher if additional sagittal knee angulation, such as knee flexion, is present. All five studies analyzing the influence of rotation combined with knee flexion (5°–15°) showed an HKA change of > 2° between 15°IR and 15°ER.

**Conclusion:**

Malrotation is frequently present on LLR, possibly influencing the measured alignment especially in knees with extension deficit. Surgeons must consider this when measuring and treating deformities (high tibial osteotomy or total knee arthroplasties), and analyzing surgical outcomes. Especially in patients with osteoarthritis with knee extension deficits or postoperative swelling, the effect of malrotation is significantly greater.

## Introduction

Long-leg radiographs (LLR) show the lower limb from the femoral head to the ankle. They are obtained to determine the mechanical axis to analyze deformities and plan deformity corrections in the frontal plane [52]. Moreover, LLR are used in complex total knee arthroplasty to plan appropriate bone resections [15, 38] and are essential for postoperative evaluation.

In the last 20 years, several studies analyzed factors that influence the measured parameters on LLR. Some claimed concerns regarding the reliability and accuracy of LLR. In daily clinical practice, limb rotation on LLR is a major topic: Is the limb properly positioned? How much limb rotation is present on the radiograph? How does it influence the measured limb alignment? Furthermore, there is no consensus on how much limb rotation can be accepted and how it influences treatment selection and surgical planning. Moreover, in clinical practice, surgeons must decide whether LLR should be repeated due to the presence of limb malrotation determined between additional radiation exposure for the patient and measurement inaccuracy.

Therefore, this systematic literature review aimed to provide an overview of studies that discussed lower limb rotation on LLR to describe how limb rotation can influence anatomic and mechanic limb alignment measurements alone and in combination with knee flexion or varus/valgus alignment; and the impact on diagnosis, treatment selection, surgical planning, or surgical outcomes. We hypothesized that axial limb rotation influences the measured limb alignment and that there is a clinical impact in treatment planning or decision making.

## Materials and methods

A literature search was conducted in June 2021 using the MEDLINE (PubMed), Web of Science (Clarivate Analytics), Cochrane Library, and EMBASE databases. The following search term was used: ((radiograph OR X-ray) AND (position OR rotation) AND limb alignment). References were managed using EndNote X9.3.

Inclusion criteria for studies were as follows:

- Studies published between 1989 and June 2021.
- Experimental studies (using either specimens or synthetic bones), clinical studies (prospective or retrospective using radiographs of patients), and systematic reviews.
- Studies that analyzed the influence of limb rotation on anatomic and mechanical alignment measurements.

Exclusion criteria for studies were as follows:

- Conference abstracts, editorial letters, and book chapters.
- Theoretical approaches.
- Studies published in languages other than English.
- Studies that analyzed the influence of rotation on only one bone (the tibia or femur).
- Studies that measured tibiofemoral alignment, but not its measurement variability.
- Studies that did not refer limb rotation measurements to the measured limb alignment.
- Studies that measured the change of alignment influenced by a surgery (e.g., component position after total knee arthroplasties (TKA)], but not by limb rotation.

**Fig. 1:**
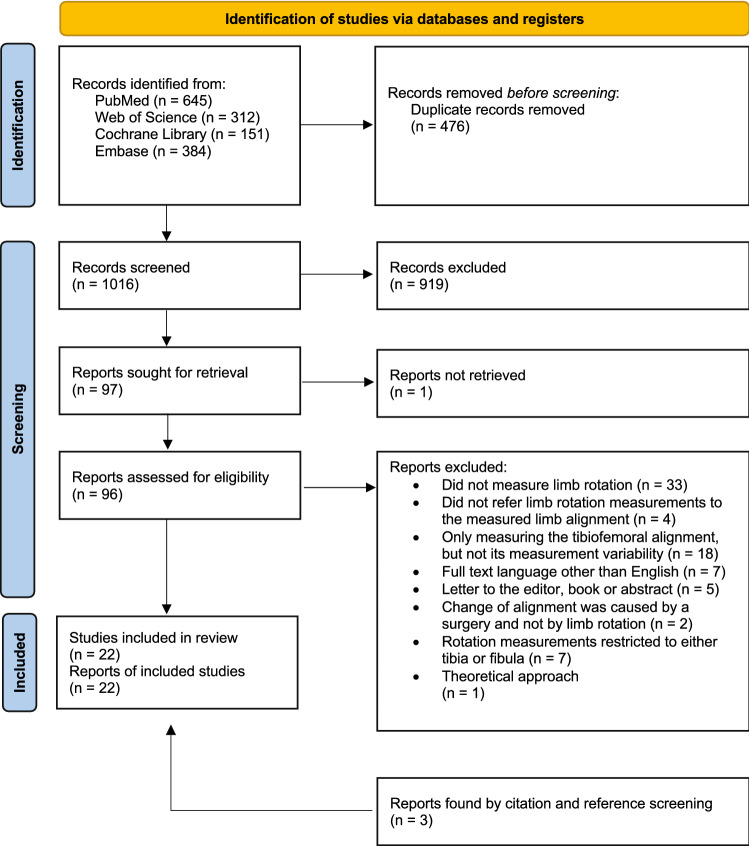
Flowchart according to the PRISMA guidelines

All steps for the identification of studies (record identification, record screening, report eligibility assessment, and report exclusion and inclusion) were independently carried out by two reviewers. Any differences regarding the included studies between the two reviewers were discussed and a consensus was reached. References and citations of the finally included articles were scanned for additional relevant articles matching the abovementioned inclusion criteria (Fig. 1).

### Interpretation and analysis of the included articles

**Fig. 2:**
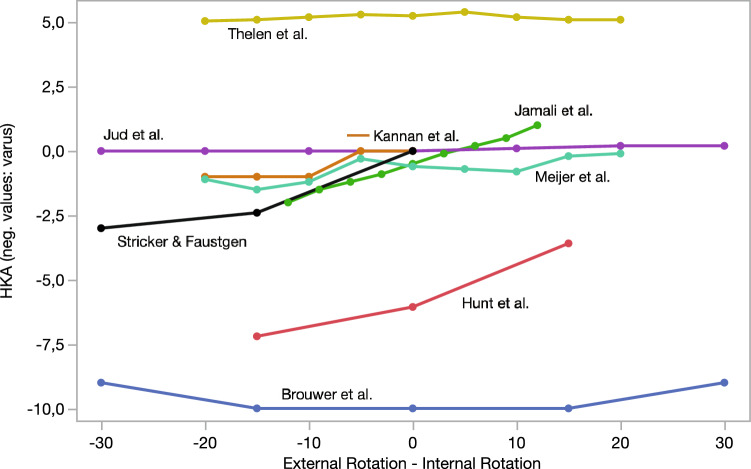
Comparison of studies reporting on the influence of rotation on the HKA. (There was no distinction between mean and median values. Some studies reported on the change from the HKA to neutral rotation. Some presented absolute values but were adapted to HKA change compared to the neutral rotation for better illustration [55]. Values of Thelen et al.[58] Meijer et al. [40], and Jamali et al. [21] were obtained from graphs of their publications.)

**Fig. 3:**
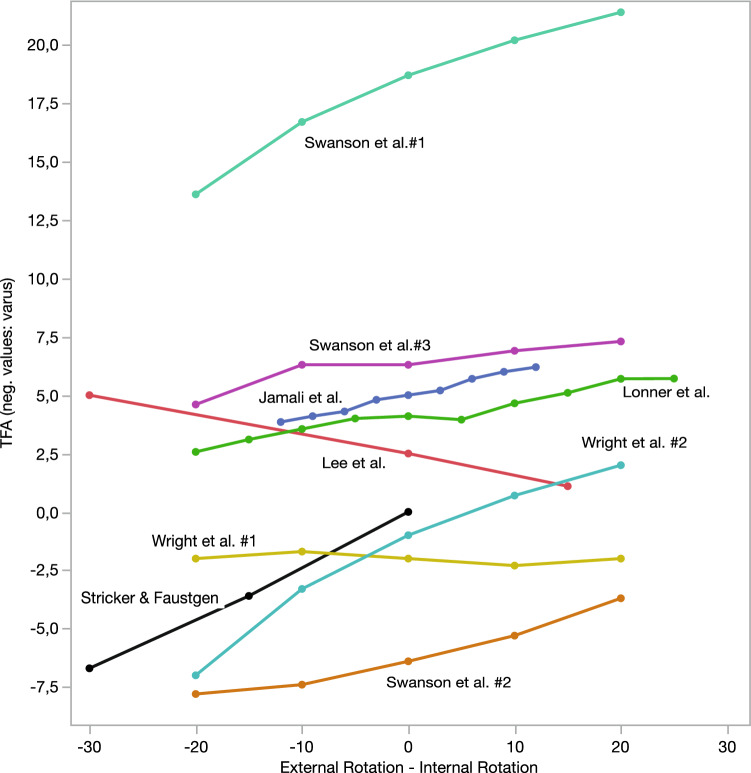
Comparison of studies reporting on the influence of rotation on the FTA. (#: different objects of the study. Some studies reported on the change of the TFA compared to neutral limb position. Some presented absolute values but were adapted to TFA change compared to the neutral rotation for better illustration [55]. There was no distinction between mean and median values. Values of Lonner et al.[35] and Jamali et al.[21] were obtained from graphs of their publications.)

**Fig. 4:**
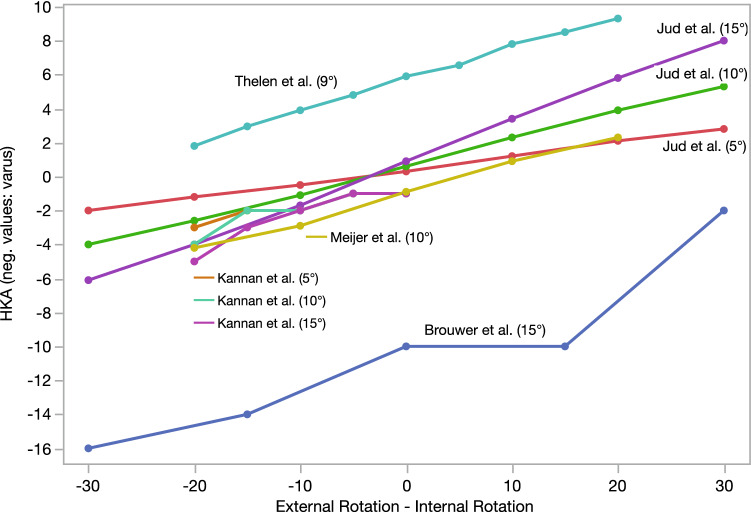
Comparison of studies reporting on the influence of rotation in combination with knee flexion on the HKA. (Some studies reported about the change in the HKA compared to neutral limb position. Some presented absolute values. Values of Thelen et al. [58] were obtained from graphs of their publications.) Values in brackets behind the study name reflect knee flexion

Relevant data from the included articles were extracted and recorded in a separate table designed a priori. Descriptions of the included studies were summarized, simplified, and grouped for syntheses and comparison to answer the research aims. Publishing year, study type, study aim, sample size, sample, image modality, and analyzed alignment measurements of the included studies are summarized in Table 1. A short description of the methods, and main findings of the included studies are summarized in Table 2 in “Appendix”. The included studies were descriptively compared. If applicable and present, descriptive statistics (means ± standard deviations; median, ranges) were presented for each study. The comparison between the results of studies analyzing the influence of rotation on anatomic tibiofemoral angle (TFA) and those of studies analyzing the influence of rotation on the hip-knee-ankle angle (HKA) is visually displayed in Figs. 2, 3, and 4. No assessment or comparison of the study quality was comprehensively performed because study designs, methods, and purposes of the studies varied widely. Nevertheless, the main limitations and methodological differences of the included studies are described in the Discussion section and summarized in Table 2 in “Appendix”. Due to the high heterogeneity among studies, no meta-analysis was performed and only descriptive comparisons were performed.

**Table 1:** Description of the included studies

References	Year	Aim	Study type	Sample size and test object	Image modality	Measured alignment parameters
Brouwer et al. [8]	2007	1. Effect of rotation (and flexion) on alignment	Experimental	1 specimen (leg) without soft tissue	LLR (tube at a distance of 150 cm)	HKA
Hunt et al. [19]	2006	1. Effect of rotation (foot) on alignment	Prospective	19 Lower limbs of 10 patients	wbLLR (distance X-ray source–knee: 8 feet)	HKA, MAD
Jamali et al. [21]	2017	1. Effect of rotation on alignment	Retrospective	30 Patients	3D models based on CT scans (supine position)	TFA, HKA, AMA, aLDFA, mLDFA, mMPTA
Jud et al. [24]	2019	1. Effect of rotation (and flexion, varus/valgus) on alignment	Prospective	7 Patients (coronal alignment from 9° varus to 9° valgus)	3D surface models generated from CT scans (supine position)	HKA
Kannan et al. [25]	2012	1. Effect of rotation (and flexion) on alignment	Experimental	1 Synthetic tibia and femur	LLR (distance X-ray source—model: 30 cm)	HKA, mMPTA, FFA
Kawahara et al. [26]	2020	1. Effect of rotation (and flexion) on alignment 2. Clinical impact (knee osteotomy simulation)	Retrospective	51 Varus osteoarthritis patients who underwent HTO	Digitally reconstructed radiography images reconstructed from preoperative CT	WBL, mMPTA, planned opening angle of the HTO
Kawakami et al. [27]	2004	1. Effect of rotation (and flexion) on alignment 2. Clinical impact (knee osteotomy simulation)	Prospective	31 Varus limbs of 20 patients	wbLLR and 3D models based on helical CT scans	TFA, HKA
Kendoff et al. [28]	2007	1. Effect of rotation (and flexion) on alignment	Experimental	4 Specimens (8 limbs)	Image-free navigation	HKA measured with a navigation system
Khare and Jaramaz [29]	2017	1. Effect of rotation (foot) on landmarks and alignment	Experimental	10 Specimens (hip to toe)	CT images (supine)	Different anatomic landmarks, femoral and tibial mechanical axis
Lee et al. [33]	2013	1. Effect of rotation (foot) on alignment	Prospective	40 Patients (80 limbs) with genu varum and medial knee pain	wbLLR and a.p. short-plane knee radiographs	TFA, WBL, ratio of weight-bearing line/tibial plateau width
Lonner et al. [35]	1996	1. Effect of rotation (and flexion) on alignment	Experimental	1 Synthetic femur and tibia surfaced with a TKA	Short-plane a.p. radiographs	TFA, tibial component alignment
Maderbacher et al. [36]	2017	1. Effect of rotation on alignment 2. Clinical impact (misinterpretation of TKA alignment)	Retrospective	100 Patients with TKA treatment	wbLLR (distance X-ray source—cassette: 180–200 cm	HKA, tibial and femoral component alignment
Maderbacher et al. [37]	2019	1. Effect of rotation on alignment	Retrospective	100 Patients	wbLLR	HKA, AMA, mLPFA, mLDFA, mLDTA, mMPTA
Meijer et al. [40]	2016	1. Effect of rotation (deformity, flexion) on alignment	Experimental	1 Synthetic limb with a TKA	Radiographs taken with EOS	HKA
Moon et al. [42]	2020	1. Effect of rotation (and flexion) on alignment	Retrospective	90 Patients (180 limbs)	wbLLR and radiographs taken with EOS	HKA
Radtke et al. [47]	2010	1. Effect of rotation on alignment	Experimental	1 Synthetic femur and tibia surfaced with a TKA	LLR (distance X-ray source-object: 210 cm)	AMA, mLPFA, mLDFA, mMPTA mLDTA
Sabharwal et al. [49]	2008	1. Effect of rotation on alignment	Retrospective	29 Patients (31 limbs)	wbLLR (tube distance at least 203 cm)	MAD
Stricker and Faustgen [55]	1994	1. Effect of rotation on alignment 2. Clinical impact (misdiagnosis of Blount’s disease)	Prospective	17 Children (33 limbs) with bilateral bowleg deformity (18–36 months)	wbLLR	HKA, TFA, MDA
Swanson et al. [56]	1999	1. Effect of rotation (and varus/valgus) on alignment 2. Clinical impact (knee osteotomy simulation)	Experimental	3 Synthetic tibia and femurs	LLR (distance X-ray source-model: 92 inches)	TFA, AMA
Thelen et al. [58]	2012	1. Effect of rotation (and flexion) on alignment	Experimental	3 Synthetic bone models (tibia, fibula, femur)	Radiographs taken with a biplanar slot scanning system (EOS)	HKA
Wright et al. [63]	1991	1. Effect of rotation (and flexion) on alignment	Experimental	2 Above-knee amputated limbs	a.p. radiographs (35 × 90 cm)	TFA
Yoo et al. [64]	2020	1. Effect of rotation (and flexion) on alignment	Retrospective	115 Patients with TKA	Radiographs taken with EOS	HKA

Descriptions of the included studies were summarized, simplified, and focused on the aim of the present systematic review

*mLDFA* mechanical lateral distal femoral angle, *MPTA* medial proximal tibial angle, *wbLLR* weight-bearing long-leg radiographs, *LLR* non-weight-bearing long-leg radiographs, *MPTA* medial proximal tibial angle, *aLDFA* anatomic lateral distal femoral angle, *AMA* anatomical mechanical angle of the femur, *MAD* mechanical axis deviation, *FFA* frontal femoral angle, *MDA* proximal tibial metaphyseal–diaphyseal angle, *JLCA* joint line convergence angle, bowing angle of the femur (angle between the anatomic femoral axis and proximal femoral shaft), *WBL* distance from medial edge of tibia plateau to mechanical limb axis in mm

**Table 2:** Methods and results of the included studies

Study	Rotation angles, Flexion angles, further information	Main findings, conclusions, main limitations
Brouwer et al. [8]	Rotation: from 30° ER to 30° IR (15° increments) Flexion: 0°, 15°, 30°	Rotation or flexion alone did not change the HKA (-10° in NR) by more than 1°. Rotation and flexion led to large changes of the HKA: HKA -10° for 15° flexion with 15° IR, HKA -14° for 15° flexion with 15° ER; HKA 2° for 15° flexion with 30° IR, and HKA -16° for 15° flexion with 30° ER. For the varus aligned limb, flexion in combination with ER caused more changes than flexion with IR Limitations: only one specimen was analyzed
Hunt et al. [19]	Rotation: 0°, 15° IR and 15° ER of the foot	IR of the foot resulted in less measured varus alignment and ER leads to greater measured varus alignment: HKA: -7.2° ± 3.5° in 15° ER, -6.1° ± 3.8° in NR, -3.6° ± 2.7° in 15° IR; MAD: 27.5 mm ± 13.8 in 15° ER, 22.9 mm ± 14.0 in NR, 14.3 mm ± 9.8 in 15° IR Limitations: rotation is controlled by the foot position – not by the position of the patella; no information was given about the deformity in the axial or sagittal plane
Jamali et al. [21]	Rotation: from 12° ER to 12° IR (3° increments) Others: 3D models were created from the CT data. A.p. images were obtained using a rendering software	Small amount of rotation led to a statistically significant difference relative to the neutral position for all mechanical and anatomical alignment parameters except for aLDFA and mLDFA. Effects may be small and their clinical importance is unknown. For the HKA, TFA and AMA 3° rotation led to a statistically significant difference compared to the NR position. IR leads to more valgus and ER leads to more varus. AMA was 5.33° in NR, 5.4° in 12° IR, and 5.1° in 12° ER (average decrease of 0.0146° per degree of rotation) Limitations: Exact values of the change of alignment parameters were not reported; for comparison to other studies, the change of TFA and HKA due to limb rotation was extracted form a graph
Jud et al. [24]	Rotation: from 30° ER to 30° IR (10° increments) Flexion: 0°, 5°, 10°, 15°, 20°, 30° Others: For each simulation a.p. projections were calculated from the 3D surface model, and the HKA were measured	HKA is not influenced by rotation when the knee is in 0° flexion, but it is by the combination of rotation and flexion. Changes of the HKA are comparable between patients with coronal alignment between 9° varus and 9° valgus: *Rotation and 0° flexion—HKA*: Median change referenced to the HKA-baseline 0° (min./max. deviation from the median: -0.8°/0.2°) in 20° ER, 0° (-0.3°/0.0°) in 10° ER 0.1° (-0.3°/0.2°) in 10° IR, 0.2° (-0.9° /0.7°) in 20° IR. Rotation and 10° flexion – HKA: -2.6° (-1.2°/0.3°) in 20° ER, -1.1° (-0.6°/0.2°) in 10° ER, 0.6 (-0.3/0.1) in NR, in 2.3° (-0.4°/0.4°) 10° IR, 3.9° (-0.9°/0.9°) in 20° IR *Limitations*: All patients had normal sagittal alignment and femoral torsion; study is based on non-weight-bearing computer simulations
Kannan et al. [25]	Rotation: from 0° to 20° ER (5° increments) Flexion: 0°, 5°, 10°, 15°, 20°	HKA did not vary > 1° with knee flexion or rotation alone. The combination of both factors can alter the apparent HKA substantially: 10° ER, 5° flexion change of 2°; 10° ER, 10° flexion change of 2°; 20° ER, 10° flexion change of 4°. MPTA and FFA changed by ≤ 1° by flexion or rotation and ≤ 3° in combination of rotation and flexion Limitations: LLR were not performed in accordance to Paley; IR was not analyzed; only one synthetic sample was analyzed
Kawahara et al. [26]	Rotation: 0°, 5° ER and IR, 10° ER and IR Others: digitally reconstructed radiography images parallel to the surgical epicondylar axis (neutral rotation; NR), 5° and 10° external rotation (ER) or internal rotation (IR) were reconstructed from preoperative CT. Based on the images, open-wedge HTO was planned aiming for a postoperative WBL of 62.5%. The planned opening angle were measured in each image	The preoperative knee extension angle was − 2.4° ± 3.8°. Weight-bearing line (WBL) changed from 25.6 ± 10.5% in 10°ER, 26.8 ± 9.5% in NR, to 28.5 ± 9.2% in 10°IR. Differences of the WBL between the measurement at 10°, 5° ER, 5°, 10°IR and the neutral position significantly correlated with the knee flexion angle The analysis of the effect of limb malrotation on preoperative planning of open-wedge HTO showed that the weight-bearing line increased and the medial proximal tibial angle gradually increased from ER to IR, whereas the opening angle gradually decreased. As the opening angle changed only within 0.5° on average, authors concluded that malrotation of the LLR by < 10° does not influence preoperative planning in open-wedge HTO in cases without knee extension deficits. However, knees with > 10° knee extension deficit showed a difference in the measured open angle of > 1° due to rotation Limitations: Maximum and minimum values of the measured difference were not given. Alignment and flexion deficits were measured using non-weight-bearing CT imaging
Kawakami et al. [27]	Rotation: measured on LLR by superimposing 3D bone models Flexion: obtained from a lateral view and calculated by the computer software Others: LLR and 3D Models were obtained using a CT. A computer software was developed to calculate TFA and HKA, its changes due to rotation, to measure knee flexion and simulate knee osteotomy from the 3D bone model. Closed-wedge HTO was simulated with a target of 4° HKA postoperative	The mean limb rotation on radiographs was 7.4 ± 3.9° of IR (range: 8° ER to 14° IR). The mean TFA and HKA on a.p. standing radiographs was 183.5 ± 5.5° (range: 178–205) and -9.2 ± 5.6° (range: -30 to -4). The mean change of HKA was 1.6° ± 1.3° (0.2–4.9) and of the TFA 3.5° ± 2.2° (0.4–8.6°). No significant correlation was found between frontal alignment on AP radiographs and the change in alignment with limb rotation. The change in HKA and FTA with limb rotation increased as the knee flexion increased Rotation may affect measurement of lower limb alignment for knee osteotomy: With IR and ER by 5°, 10°, and 15° of the osteotomy plane, the FTA changed by 0.2 ± 0.1°, 0.4 ± 0.3°, and 0.8 ± 0.4° and the HKA changed by 0.2 ± 0.1°, 0.4 ± 0.2°, and 0.7 ± 0.3°. HKA and TFA after virtual closed-wedge tibial osteotomy were measured of a case with a rotational error of the osteotomy plane. The proximal cutting surface was reattached to the distal cutting surface. The mean change in TFA was 1.6 ± 0.6°, 3.2 ± 1.1°, and 4.8 ± 1.7° for 5°, 10°, and 15° IR and ER errors in reattaching the osteotomy plane. The mean change in HKA was 0.9° ± 0.5°, 1.9 ± 1.0°, and 2.8 ± 1.5° with an internal and external 5°, 10°, and 15° osteotomy plane rotation Limitations: transverse images were obtained using a helical CT scanner while the patient was in supine position; only varus knees were included
Kendoff et al. [28]	Rotation: 0°, 5° ER and IR, 10° ER and IR, maximal rotation Flexion: 0°, 5°, 10°, 20° Others: The mechanical leg axis and the axis deviations were assessed using a navigation system	In full extension, axis measurement deviations ranging from 0.4° to 4.3° with larger deviations occurring with increasing rotation (IR or ER): mechanical axis deviation with full extension 10° IR 1.5°, 10° ER 2.1°, maximal IR 4.3°, and maximal ER 3.9°. When the knee was flexed, larger deviations were found: 5° flexion with 5°IR led to alignment errors of 3.4°, and with 5°ER to 2.8°. Over- and undercorrection might occur Limitations: measurement deviation was measured, not the absolute mechanical axis; coronal plane alignment was measured with a navigation system
Khare and Jaramaz [29]	Rotation: 0°, 10° IR, 10° ER Others: Digitally reconstructed radiographs were obtained from CT scans. Landmarks were manually identified in the radiographs and the 3D CT scans were compared and variations of the positions were measured	Rotation leads to increased errors in certain landmarks (as large as 13.1 mm for the femoral knee center and 13.6 mm for the lateral malleolus). Despite of a large variation in the measurement of landmark points, the estimation of tibial and femoral mechanical axes did not suffer inaccuracy (maximum error of 1.46° for the femoral mechanical axis and 0.66° for the tibial mechanical axis) Limitations: No weight-bearing condition and radiographs were obtained by digitalized radiographs from a CT scan
Lee et al. [33]	Rotation: 0°, 15° IR an 30° ER of the foot	Foot position in ER shows less varus and IR shows more varus. The weight bearing line shifted laterally in the 30° position and shifted medial in the 15° internal position compared to the neutral position (1.8 mm lateral, 0.2 mm medial in the LLR, and 3.5 mm lateral and 3 mm medial in the local radiograph). Results of the ratio of weight-bearing line/tibial plateau width were similar to those of the weight-bearing line Limitations: the limb was positioned by the foot position; only standard deviations were presented to report the variation from the mean
Lonner et al. [35]	Rotation: from 20° ER to 25° IR (5° increments) Flexion: 0°, 10°	ER causes less apparent anatomic valgus and IR pretends more valgus. Even in a well-aligned TKA, limb positioning will alter alignment measurements, making objective evaluation difficult: Rotation and 0° Flexion: 2.6° TFA in 20° ER, 5.7° in 25° IR; Rotation and 10° Flexion: 2.3° in 20° ER, 6.7° in 25° IR. Tibial alignment changed with 10° flexion ranging from -*5°* varus in 20°ER to 3° valgus in 25°IR Limitations: mechanical alignment was not measured; non-weight-bearing condition; short-plane radiographs; for comparison to other studies, the change of TFA due to limb rotation was extracted from a graph
Maderbacher et al. [36]	Rotation: rotation errors were calculated by the tibial-fibular overlap using a formula [38] Flexion: for all radiographs full extension and 10° flexion were assumed Others: The impact of observed rotational errors was calculated using data of previous studies: for each degree of rotation, the HKA changed in extended knees by 0.0697° and with 10° flexion by 0.0986; femoral and tibial component alignment changed by 0.0824 and 0.0504. Malalignment was defined as a deviation of femoral or tibial component alignment > 2° from 90° or deviation > 3° from neutral HKA (0°)	Limbs were positioned in average in 8.1° ± 9.3° IR (range: 36° IR and 16° ER). Mean measurement errors due to rotation were quite small. However, large ranges of wrong measurements were found indicating significant and clinically relevant measurement errors in some radiographs Mean differences of HKA between measured alignment before and after a mathematical rotational correction were -0.6° ± 0.6° ranging from -2.5° to 1.1° (0° knee flexion) and -0.8° ± 0.9° ranging from − 3.5° to 1.6° (10° knee flexion). 11 out of 100 patients were wrongly assigned to either mal- or well-aligned, which was defined as HKA within ± 3° varus or valgus Limitations: see below (Maderbacher et al. [37])
Maderbacher et al. [37]	Rotation: rotation errors were calculated by the fibular overlap using a formula [38] Flexion: for all radiographs full extension and 10° flexion was assumed Others: The impact of observed rotational errors on alignment was calculated using data of previous studies: for each degree of rotation AMA changed by 0.0558, mLPFA by 0.1892, mLDFA by 0.0824, mMPTA by 0.0504, mLDTA by 0.2185, HKA in extended knees by 0.0697° and with 10° flexion by 0.0986	Malrotation is regularly present in LLR with average rotation of the limb by -8.0 ± 9.0° IR (range: 29.4° IR and 22.1° ER). Measured alignment parameters before and after rotational correction showed the following mean differences: HKA in full knee extension: 0.6° ± 0.6° (-1.5° to 2.1°); HKA in 10° knee flexion 0.8° ± 0.9° (-2.2° to 2.9°); AMA 0.4° ± 0.5° (-1.2° to 1.6°); mLPFA 1.5° ± 1.7° (-4.2° to 5.6°); mMPTA 0.4° ± 0.5° (-1.1° to 1.5°); mLDFA 0.7° ± 0.7° (-1.8° to 2.4°); mLDTA 1.7° ± 2.0° (-4.8° to 6.4°). As all measured parameters are influenced by malposition, correct limb rotation needs to be verified Limitations: The formula used to calculate the rotational error can predict lower limb rotation on LLR with a SD of 5°, but with IR the accuracy of calculated rotations decreases; calculated corrections are based on previous studies by Lonner, Jiang and Insall, and Radtke; these values are abstract and vary between mentioned studies. Moreover, these studies were conducted, as outlined by the present review, with small sample sizes; the study did not provide new data regarding the relationship between rotation and alignment deviation
Meijer et al. [40]	Rotation: from 20° ER to 20° IR Flexion: 0°, 5°, 10°, 15°, 20° Others: change of the tibiofemoral alignment from 15° varus to 15° valgus (5° increments)	Changes of rotation, flexion, and tibiofemoral alignment in the frontal plane alone had no major impact on the measured HKA (different rotations with 0° varus/valgus and 0° flexion changed between 0.1 and 1.5°). Changes in varus/valgus alignment and rotation with a fully extended knee did not show major differences in HKA (different rotations with 0° flexion and 10° valgus and 10° varus led to a change of HKA by 1.6°–2.1° and -1.7°–0.3). Flexion (20°) combined with rotation with a neutral alignment showed a HKA variation of up to 13.8° (range: -5.2 to 8.6°), a change of rotation and 10° flexion in a neural aligned knee led to a change of HKA between -2.3 and 4.2°. Combining all three factors (15° valgus, 20° flexion) led to maximum HKA variation of up to 16.5° (-10.5–6°). A change of rotation with 10° varus and 10° flexion led to different measured HKA between -2.0–4.9° Limitations: some values of the change of the alignment parameters were not reported; for comparison to other studies, the change of HKA due to limb rotation was extracted form graphs
Moon et al. [42]	Rotation: was assessed by measuring the deviation of the patellar center inward or outward relative to the midpoint between the femoral condyles Flexion: the knee extension and flexion was measured on the EOS images Others: HKA measurement between LLR and EOS was calculated (dHKA) Patients were divided into two subgroups on the sagittal plane (knee flexion and extension group) and into two subgroups based on the axial plane (knee internal and external rotation)	The mean value of the HKA on LLR was 1.2 ± 3.9°, knee flexion angle 5.3 ± 5.3°, and patellar rotation of 4.6 ± 4.0%. Significant correlation was found between dHKA and degree of knee flexion/extension (r = 0.368). No significant correlation was found between dHKA and the axial limb rotation. A significant linear relationship between dHKA and the knee flexion/extension in patients with a patellar rotation more than 3% was found (r^2^ = 0.257) Authors concluded that the measurement accuracy of coronal alignment on LLR was influences by knee flexion, which was significant in case of small rotation. Therefore, it would be important to check the patella position especially in patients with knee flexion Limitations: correlations were presented, but no exact values to describe the relationship between rotation and measured HKA; rotation was measured with the patella position
Radtke et al. [47]	Rotation: from 20° ER to 20° IR (5° increments) Flexion: full extension	Significant effects of rotation on the measured alignment were found: IR simulated tibiofemoral valgus and ER a varus alignment. The alignment parameters in NR were AMA 6.2°, mLPFA 97.5°, mLDFA 88.2°, mMPTA 89.4°, mLDTA 94°. From 20° IR to 20° ER AMA changed from 6.8° to 4.6°, mLPFA from 101.6° to 93.6°, mLDFA 90.6° to 86.8°, mMPTA 90.4° to 88.5°, and mLDTA from 98.9° to 90.5° Limitations: only one synthetic femur and tibia; HKA or TFA was not measured
Sabharwal et al. [49]	Rotation: gauged as the percentage of proximal fibular overlap and calculated as the percentage of the proximal fibular width that was overlapped by the adjacent tibial metaphyses, at the level of the proximal fibular physis. A large value indicates greater ER Others: 2 LLR were conducted: one before and the other after ring fixator removal. Difference in the frontal alignment measurement and the limb rotation was measured	The limb tended to be positioned in ER when the radiograph was taken with the circular fixator still attached to the extremity. This tendency for ER of the limb likely contributed to an overestimation of varus alignment. Absolute difference of the MAD between the 2 LLR was 11.5 mm for the treated limb (95%CI with fixator: -3.3 (valgus) to 15.7 mm (varus)); 95%CI without fixator: -6.9 to 10 mm). The mean absolute difference in the fibular overlap between the 2 LLR was 21% (95%CI with fixator: 24% to 48%; 95%CI without fixator: 16% to 33%). Linear regression analysis demonstrated that with an increase of fibular overlap, there was a progressive increase in the magnitude of discrepancy in the measurement of MAD between the 2 LLR Limitations: the rotation was approximated by the percentage of the proximal fibular overlap
Stricker and Faustgen [55]	Rotation: 0°, 15° ER, 30° ER	Limb malrotation can reduce the validity of HKA and TFA progressively. HKA: -27.7° in NR and increased by 2.4° in 15° ER and 3.0° in 30° ER; TFA: -24.8° in NR and increased by 3.0° in 15° ER and 6.7° in 30° ER. For MDA (average 12.8° in 0° rotation according to Levine-Drennan) unpredictable variability was noted during ER, which can lead to misdiagnosis and mistreatment of Blount’s disease Limitations: the limb positioning was controlled with ultrasound; only standard deviations were presented to report the variation from the mean
Swanson et al. [56]	Rotation: from 20° ER to 20° IR (10° increments) Others: Three deformed limbs: A 18° valgus, B 7° varus and C 7° valgus (TFA). Each model had 5 radiographs in each position With a graphic software, high tibial osteotomies were simulated for model B. Osteotomies aimed to achieve 8° valgus	The effect of rotation on measured TFA was more sensitive in varus and valgus deformed limbs than in a neutral limb. Severe valgus deformities are more sensitive to the effects of ER; severe varus deformities are more sensitive to effects of IR. TFA of model A: 13.6° in 20° ER, 16.7° in 10° ER, 18.7° valgus in NR, 20.2° in 10° IR, 21.4° in 20° IR. Model B: -7.8° in 20° ER, -7.4° in 10° ER, -6.4° in NR., -5.3° in 10° IR, -3.7° in 20° IR. Model C: 4.6° in 20° ER, 6.3° in 10° ER, 6.3° in NR, 6.9° in 10° IR, 7.3° in 20° IR. AMA varied less than 1° in all three models between 20° IR and ER A simulated osteotomy based on the measured TFA of model B in 20° IR an undercorrection occurred. Ideal correction of a varus deformity was achieved when the osteotomy was simulated based on LRR with the limb in the neutral position. Based on the measured TFA in 20°ER, an overcorrection was observed Limitations: axial rotation was assessed with the help of a goniometer
Thelen et al. [58]	Rotation: from 20° ER to 20° IR (5° increments) Flexion: 1 bone in 0°, 1 bone in 9°, 1 bone in 18° Others: Biplanar slot scanning system (EOS) took an a.p. and a lateral radiograph of the models (5° valgus) for each rotation position	The impact of rotation on the HKA is relatively small in the absence of sagittal knee angulation. An axial rotation of 10° (IR or ER) resulted in a measurement error (defined as difference between the measured HKA and the actual model alignment of 5°) in the 2D a.p. view of 0.4°, 1.9°, and 3.1° and an axial rotation of 20° (IR or ER) resulted in an average measurement error of 1.4°, 4.7°, and 6.8° with 0°, 9°, and 18° of knee flexion. Summarizing the measurements of 3 observers, HKA varied from 3.8°-6.4° (model 0° flexion), 0.3–9.7° (model 9° flexion) and -0.7 – 11.8° (model 18° flexion) Limitations: only standard deviations were presented to report the variation from the mean; for comparison to other studies, the change of HKA due to limb rotation was extracted form a graph
Wright et al. [63]	Rotation: from 20° ER to 20° IR (10° increments) Flexion: 0°, 20°, 30°	Little overall difference between the rotation groups were found. The reliability of lower limb alignment measured from LLR is satisfactory. The radiographer positioned the limb within 3.1° ± 2.7° from the neutral position Rotation and 0° Flexion of the first limb: 2.0° in 20° ER, 1.7° in 10° ER, 2.0° in NR, 2.3° in 10° IR, 2.0° in 20° IR. Rotation and 0° Flexion of the second limb: 7.0° in 20° ER, 3.3° in 10° ER, 1.0° in NR, -0.7° in 10° IR, -2.0° in 20° IR. Rotation and 20° Flexion—TFA: 2.0° in 20° ER, 2.0° in NR, 0.7° in 20° IR of the first limb; 2.7° in 20° ER, 4.6° in NR, 4.0° in 20° IR of the second limb Limitations: different findings between the limbs; only two knee amputates were analyzed; only anatomical measurements were analyzed
Yoo et al. [64]	Rotation: assessed by measuring the fibula/tibia overlap, as well as the tibial and femoral component rotation from AP radiographs conducted by EOS imaging system Flexion: Flexion was measured from lateral EOS radiographs Others: correlations referring to the HKA angle were calculated	There was a significant correlation for the fibular overlap (r = 0.292)/the femoral component rotation (r = 0.317) and the HKA angle. Moreover, combined rotation and knee flexion had a greater effect on the coronal alignment than flexion/rotation alone. This was more frequently observed during the early period after TKA Limitations: Rotation was only described by the fibular overlap and the component position. Relationship between rotation/flexion was expressed by calculating correlation coefficients

Descriptions of the included studies were summarized, simplified, and focused on the aim of the present systematic review (negative values of HKA and TFA indicate varus alignment

*mLDFA* mechanical lateral distal femoral angle, *MPTA* medial proximal tibial angle, *wbLLR* weight-bearing long-leg radiographs, *LLR* non-weight-bearing long-leg radiographs, *MPTA* medial proximal tibial angle, *aLDFA* anatomic lateral distal femoral angle, *AMA* anatomical mechanical angle of the femur, *MAD* mechanical axis deviation, *FFA* frontal femoral angle, *MDA* proximal tibial metaphyseal–diaphyseal angle, *JLCA* joint line convergence angle, bowing angle of the femur (angle between the anatomic femoral axis and the proximal femoral shaft), *WBL* distance from medial edge of tibia plateau to mechanical limb axis in mm, *NR* neutral rotation, *IR* internal rotation, *ER* external rotation

## Results

### Study selection

A total of 22 studies, including 19 studies from the databases and three studies [26, 28, 55] from the citations of the included studies, were selected based on the inclusion criteria and reviewed. Figure 1 presents the study selection process according to the PRISMA guidelines. Some studies met the inclusion criteria but were excluded. For example, Oswald et al. [43] and Jiang and Insall [22] measured the influence of rotation only on the femoral alignment. Some studies (e.g., [14, 32]) were excluded because the limb rotation was either not measured or only approximated. Other studies [2, 30, 31, 38] changed or measured the limb rotation but did not analyze its influence on tibiofemoral alignment.

### Study characteristics: image modalities, selected subjects, and radiographic measurements

A large heterogeneity was found among the included studies in terms of image modalities, subjects, and analyzed alignment measurements (Table 1). The included studies used different image modalities as follows: short-plane anteroposterior radiographs [35, 63], weight-bearing LLR [19, 36, 37, 49, 55], LLR without weight bearing [8, 25, 47, 56], computed tomography (CT) [21, 24, 26, 29], navigation system [28], EOS imaging [40, 58, 64], weight-bearing LLR combined with EOS imaging [42], weight-bearing LLR and a helical CT [27], and short-plane knee radiographs and LLR [33].

Of the 22 studies included, four studies used post-mortem specimens or amputated limbs [8, 28, 29, 63] and six studies used synthetic bone models [25, 35, 40, 47, 56, 58]. Except Khare and Jaramez [29] (*n* = 10), these studies used less than five specimens or test objects [8, 25, 28, 35, 40, 47, 56, 58, 63].

Three studies used CT data from patients [21, 24, 26, 27] and five studies used radiographs from patients retrospectively [36, 37, 42, 49, 64]. Hunt et al. [19], Stricker and Faustgen [55], and Lee et al. [33] obtained radiographs from patients with different rotation positions prospectively. The sample size ranged from 9 [24] to 115 [64].

The included studies used different radiographic parameters. The TFA was frequently used to analyze anatomic alignment [21, 27, 35, 55, 56, 63] and HKA was the most frequently used to analyze mechanical alignment [8, 19, 21, 24, 25, 27, 28, 40, 42, 55, 58, 64].

### Influence of rotation on alignment measurements

The materials and methods and results of the studies are summarized in Table 2 in “Appendix”. Several studies reported that ER causes less apparent valgus and leads to more varus and IR causes more apparent valgus and leads to less varus [19, 21, 26, 35, 47, 49, 56]. In contrast, Lee et al. [33] described that the foot position (0°, 15° IR, and 30° ER) in 80 lower limbs of 40 patients had little effect on alignment measures on LLR. An externally rotated foot position showed less varus, and an internally rotated position showed more varus.

There is no consensus to which extent rotation can influence alignment measures. Differences in the magnitude between the relationship of limb rotation and HKA are shown in Fig. 2. We found four studies [19, 21, 28, 55] describing an average change of > 2° HKA between 15° IR and 15° ER in a fully extended leg. A smaller change in HKA between 15° IR and 15° ER was found in four studies [8, 24, 40, 58]. For example, Jud et al. [24] found in seven patients that 10° IR led to a median change of the HKA by 0.1° (minimal deviation from median, − 0.3°; maximal deviation from median, 0.2°), 20° IR leads to 0.2° (− 0.9°; 0.7°), 10° ER leads to 0.0° (− 0.3°; 0.0°), and 20° ER leads to 0.0° (− 0.8°; 0.2°). In contrast, Hunt et al. [19] described that varus HKA decreased from 6.1° ± 3.8° in neutral position to 3.6° ± 2.7° at 15° IR and increased to 7.2° ± 3.5° at 15° ER. Additionally, studies using patient data reported a significant change of HKA due to malrotation [27, 36, 37]. For example, Kawakami et al. [27] described a mean change of HKA of 1.6° ± 1.3°, ranging from 0.2° to 4.9°, due to malpositioning on the radiographs. Jamali et al. [21] found that even a limb position of 3° variation compared to the neutral position leads to a statistically significant difference of the measured HKA and TFA. However, the authors stated that these effects may be small, and their clinical importance is unknown [21]. The relationship between anatomical limb alignment (TFA) and limb rotation of included studies is shown in Fig. 3.

Lonner et al. [35] reported TFA changes from 2.57° valgus in 20° ER to 5.71° valgus in 25° IR. Kawakami et al. [27] found a mean change of TFA of 3.5° ± 2.2° (range 0.4°–8.6°) due to malrotation of the patient’s limb on LLR. Lee et al. [33] reported a decrease in the femoral–tibial angle (FTA) in short-plane radiographs from 2.5° ± 4.3° in neutral position to 5° ± 5.2° at 30° ER of the foot and 1.1° ± 5.0° at 15° IR of the foot. Wright et al. [63] analyzed two amputated limbs. They concluded that the TFA showed only small effect on rotation with the fully extended limb rotated < 10°. The alignment of the first limb changed from 1.7° to 2.3° valgus from 20° ER to 20° IR, but the alignment of the second limb changed from 7.0° valgus to − 2.0° varus from 20° ER to 20° IR. Swanson et al. [56] found that the TFA changed from 6.4° varus in the neutral position to 5.3° and 3.7° in 10° and 20° IR and 7.4° and 7.8° in 10° and 20° ER. Other alignment angles were analyzed, for example, by Radtke et al. [47]. From 20° IR to 20° ER, AMA changed from 6.8° to 4.6°, mLPFA from 101.6° to 93.6°, mLDFA 90.6° to 86.8°, mMPTA 90.4° to 88.5°, and mLDTA from 98.9° to 90.5°.

### Influence of rotation in combination with knee extension deficits or varus–valgus alignment

12 studies analyzed the influence of rotation in combination with knee flexion [8, 24–28, 35, 40, 42, 58, 63, 64]. There is consensus that the impact of rotation on the mechanical alignment is higher, if an additional sagittal knee angulation, such as knee flexion, is present [8, 24–28, 35, 40, 42, 58, 63, 64]. Figure 4 presents studies on the combination of rotation and knee flexion between 5° and 15°. All five studies analyzing the influence of rotation in combination with knee flexion (5° to 15°) showed a HKA change of > 2° between 15° IR to 15° ER [8, 24, 25, 40, 58]. Furthermore, Kendoff et al. [28] described alignment deviation from the initial leg alignment of 3.4° with 5° IR and 5° knee flexion and 2.8° with 5° ER and 5° knee flexion. Kannan et al. [25] found that the HKA did not vary by > 1° when their saw bone model was flexed from 0° to 20° in neutral rotation position or when the model was externally rotated up to 20° in full extension. Greater variation was found when flexion and rotation were combined: 10° ER and 5° flexion led to a change in HKA by 2° and 20° ER and 10° flexion led to a change of HKA by 4°. Meijer et al. [40] reported that a change of rotation from 20° IR to 20° ER in a neutrally aligned, 10°-flexed knee leads to a change of HKA measurement of up to 6.5° (measurement difference between − 2.3 and 4.2°). Thelen et al. [58] found that axial rotation of 10° (IR or ER) of their lower limb model resulted in HKA deviations of 0.4° with full knee extension, 1.9° with 9° knee flexion, and 3.1° with 18° knee flexion. Brouwer et al. [8] showed that the combination of flexion and rotation leads to large changes in HKA. For example, 15° knee flexion with 15° ER led to a change in the HKA measurement of 4°. Jud et al. [24] reported that 10° knee flexion in combination with 10° IR and 20° IR led to 2.3° valgus and 3.9° valgus and 10° ER and 20° ER led to − 1.1° varus and − 2.6° varus.

The influence of deformity and malrotation on measured alignment remains controversial. Brouwer et al. [8] revealed that, in case of a varus deformity, flexion in combination with external rotation causes more changes than flexion in combination with internal rotation. Stricker and Faustgen [55] analyzed 17 children with bowleg deformity and concluded that limb malrotation can reduce the validity of HKA and TFA progressively. They found that HKA was 27.7° at neutral rotation and significantly increased by 2.4° with 15° ER and 3.0° with 30° ER. The TFA was 24.8° at neutral rotation and significantly increased by 3.0° with 15° ER and 6.7° with 30° ER. Swanson et al. [56] pointed out that the effect of malrotation on measured TFA is more sensitive in varus and valgus deformed limbs than in neutral aligned limb. Moreover, severe valgus deformities are more sensitive to the effects of ER, and severe varus deformities are more sensitive to the effects of IR. In contrast, Jud et al. [24] found, in seven patients with coronal alignment between 9° varus and 9° valgus and no further deformity, that the change in the HKA induced by knee flexion and malrotation had almost equal influence on the HKA. Kawakami et al. [27] found no correlation between the degree of varus alignment on AP radiographs and change in alignment with limb rotation. The flexion of the knee during radiography was the main source of the effect of rotation on limb alignment measurements. Meijer et al. [40] investigated the influence of varus/valgus alignment, limb rotation, and knee flexion in a synthetic bone model surfaced with a TKA. They found that a change in varus/valgus alignment from 15° varus to 15° valgus and a change in the rotation from 20° IR to 20° ER with a fully extended knee did not show major differences in HKA. However, rotating a fully extended knee with 10° valgus or 10° varus led to an average change of 1.8° (range between 1.6° and 2.1°) or 0.7° (range between − 1.7° and 0.3°) in HKA. Maximum measurement differences were found if all three factors (knee alignment, limb rotation, and knee flexion) were combined. For example, 10° varus with 10° flexion with rotation changes from 20° IR and 20° ER led to difference of − 2.0 to 4.9° in HKA.

### Studies analyzing the impact on clinical decision making

The change in anatomical and mechanical alignment due to limb rotation and its impact on treatment selection, surgical planning, and surgical outcome are only analyzed by few studies.

Maderbacher et al. [36] found, in average, only small rotation-related measurement errors of HKA (− 0.6°). However, large ranges of wrong measurements were found (− 2.5 valgus to 1.1 varus), indicating significant and clinically relevant measurement errors in some radiographs. Moreover, 11% of TKAs were wrongly assigned to either maligned or well aligned, which was defined as HKA within ± 3° varus or valgus. Moreover, Stricker and Faustgen [55] pointed out that malrotation on radiographs can lead to misdiagnosis and mistreatment of Blount’s disease.

Results of a computer-simulated HTO performed by Swanson et al. [56] demonstrated the influence of limb rotation on HTO planning. Osteotomy planning based on the measured TFA with limb rotation resulted in undercorrection with a limb rotated in 20° IR and resulted in overcorrection in 20° ER of the limb.

Kawahara et al. [26] analyzed the effect of limb malrotation on preoperative planning of open-wedge HTO in 51 knees. Digital radiographs were reconstructed from CT datasets in neutral position, 5° IR and ER, and 10° IR and 10° ER. The weight-bearing line percentage, medial proximal tibial angle, and planned opening angle were measured. The preoperative weight-bearing line (25.6 ± 10.5% in 10°ER to 28.5 ± 9.2% in 10°IR) and medial proximal tibial angle gradually increased from ER to IR, whereas the opening angle gradually decreased. As the opening angle changed only within 0.5° on average, the authors concluded that malrotation of the LLR by < 10° does not influence preoperative planning in open-wedge HTO in patients without knee extension deficits. In patients with knee extension deficits of > 10°, the opening angle remarkably differed by > 1°.

Other studies judged their findings as clinically relevant or irrelevant without applying their results to a clinical setting. For example, Thelen et al. [58] considered the abovementioned HKA deviation error of 1.9° in the 9° knee flexion position and 3.1° in the 18° knee flexion position as clinically relevant.

## Discussion

The main findings of this systematic review are that external limb rotation causes less apparent knee valgus and leads to more varus. Internal limb rotation causes more valgus and leads to less varus [19, 21, 26, 35, 47, 49, 56]. However, there is no consensus on the influence of limb rotation on alignment measures. The impact of rotation on mechanical alignment is higher if additional sagittal knee angulation, such as knee extension deficits, is present [8, 24–28, 35, 40, 42, 58, 63, 64]. All studies that analyzed the influence of rotation combined with knee flexion (5° to 15°) on HKA found a change by > 2° between 15° IR to 15° ER [8, 24, 25, 40, 58]. This has to be considered especially for patients with osteoarthritis before HTO or TKA with extension deficits [26].

Malrotation of the limb is frequently present on LLR [14]. For example, Kawakami et al. [27] found that 31 limbs on LLR were in average 7.4° ± 3.9° (range, 8° ER to 14° IR) internally rotated. In two studies with 100 LLR, the limb position was found to be in 8.1° ± 9.3° IR, ranging from 36° IR to 16° ER [36], and 8.0° ± 9.0° IR ranging from 29.4° IR to 22.1° ER [37]. It can be assumed, that in patients with severe deformities or circular fixator, malpositioning is observed more frequently and to a greater extent [49].

However, the assessment of malrotation and the interpretation of the quality of the radiograph can be difficult [26, 31]. The detection of limb rotation is important to avoid alignment measurement errors. Different methods have been used to approximate limb rotation. For example, the overlap of the proximal fibula at the tibia was used to determine the rotation in comparison to previous radiographs [14, 49, 55, 64] or even calculate the rotation with the help of a formula according to Maderbacher et al. [38]. Moon et al. [42] suggested to use the position of the patella relative to the width of the femoral condyles in the frontal plane. However, this method is likely inaccurate in patients with patellar disorders. Radtke et al. [47], Yoo et al. [64], and Kim et al. [30] used a ratio between visual landmarks and distances of TKA components. The ratio is dependent on the TKA type. Radiographic imaging guidelines defined in the clinic were shown to enhance the quality of radiographs [4, 62]. Tools to improve limb positioning, such as positioning jigs or radiographic markers, could reduce the number of malrotated radiographs [2, 16, 49].

Malrotation can lead to a three-dimensional change in the positions of anatomical landmarks [29] and misinterpretation of the alignment in postoperative radiographs [31]. Several studies reported that ER causes less apparent valgus and leads to more varus. IR causes more valgus and leads to less varus [19, 21, 26, 35, 47, 49, 56]. However, Lee et al. [33] described that the foot position in ER shows less varus and IR position shows more varus. We found no consensus to which extent rotation can influence alignment measures. The majority of studies reported that knee flexion without limb rotation has only a small effect on measured alignment without limb rotation. However, Koshino et al. [32] analyzed 17 healthy knees and described that a knee flexion deformity leads to underestimation of varus angulation of the knee.

With or without knee flexion, the degree of malrotation and its influence on alignment measurements are discussed controversially. Differences between the findings of the studies can be explained by the large heterogeneity of their methods. The included studies comprised radiological testing with amputated limbs, specimens, or synthetic bones and prospective and retrospective analyses of radiographs with patients. The findings of radiographic tests are often difficult to apply to the clinical settings. In contrast, in the LLR of patients, knee flexion often cannot be determined, and the amount of rotation is estimated using the TKA component, fibular overlap, or patellar position [36, 42, 64]. Moreover, the applied imaging modality differed using CT data, short-plane radiographs, or LLR with differences in the distance between the X-ray source and the knee or object. This might alter the results [1, 35, 45, 51]. Moreover, the neutral position of the limb was defined differently (e.g., overlap of the femoral condyles [58], patella straight forward [24]), which leads to different limb rotations on radiographs [7]. Moreover, the applied methods to set the rotation of the test object or limb were different, potentially lacking accuracy and comparability. For example, to control limb rotation, studies used the foot position [19, 33] or ultrasound to overlap the posterior femoral condyles [55]. Other studies controlled axial rotation with the help of a goniometer [56] or created three-dimensional (3D) models from CT data, rotated the limb around the virtual longitudinal axis, and obtained anteroposterior images for the different positions using a rendering software [21].

Included studies using the synthetic bone have only used a small sample size [8, 25, 35, 47]^63^,^56^,^ 58^ and do not depict the anatomic variability between subjects due to sex, age, osteoarthritis, or ethnicity [18, 34, 39, 65]. These studies might underestimate the influence of malrotation in an individual. Synthetic bones often have, if present, only a deformity in the frontal plane. The influence of rotation on alignment parameters was not analyzed with concomitant deformities. Especially, varus deformity of the knees with osteoarthritis is associated with rotational deformity as the tibia and the femur tend to rotate externally in relation to the hip and ankle [65]. These concomitant deformities, other than in the frontal plane, were usually not reported by patient-dependent studies.

Moreover, studies reporting on only a small influence of rotation on alignment parameters mostly judged their findings in consideration of the mean difference in the neutral limb position. The exact values or maximal deviations in studies with more objects were not commonly reported, and only correlation coefficients between rotation and alignment parameters [42, 64] or standard deviations were reported to indicate the variation from the mean [21, 26, 33, 55, 58] However, minimum and maximum measurement deviations should be considered [36, 37, 40, 55, 63]. For example, one of the most cited studies on limb rotation on radiographs [63] was conducted with two above-knee amputated limbs. The FTA of the first limb did not change from 20° ER to 20° IR. However, the alignment of the second limb changed by 4° within 10° IR and 10° ER and by 9° from 20° IR to 20° ER. Despite the differences between both limbs, the authors concluded that, if the limb was rotated < 10° from neutral rotation, this rotation did not have a significant effect on the anatomical alignment with the limb rotated < 10°. In our point of view, more research is needed to clarify the relationship between rotation and alignment measurements and determine reasons for different magnitudes of this relationship found between studies and for some individuals.

Additionally, data of the included studies are presented differently. For example, the relationship between rotation and measured anatomical and mechanical alignment is presented in a figure [21, 58], a regression model [47], or by reporting the deviation from the neutral position for specific rotation positions. Therefore, the syntheses of these studies in a mathematical systematical approach were not possible. We depict the reported values in Figs. 2, 3, and 4 as accurate as possible. Maderbacher et al. [36, 37] were not included in the figures as they did not provide new data regarding the relationship between rotation and alignment deviation. They applied an interesting approach and transferred the findings of previous radiographic tests [22, 35, 47] to a clinical setting. They found large ranges of wrong measurements, indicating significant and clinically relevant measurement errors in some radiographs. However, their study’s results are limited by the used mathematical approach to determine limb rotation and may deviate from the true relationship between rotation and changes in alignment, which is emphasized by this systematic review as different studies report about the differences of this relationship.

Only few studies analyzed the implication of malrotation on radiographs in clinical practice. Furthermore, the interpretation of the findings with respect to clinical settings is often judged differently. Swanson et al. [56] concluded that already a 10° ER or IR can already cause variation in the measured TFA by several degrees, and therefore, inaccurate TFA measurements can lead to wrong preoperative osteotomy planning and can contribute to poor postoperative outcomes. In osteotomies in varus knees, malrotation resulted in an undercorrection in 20° IR and overcorrection in 20° ER [56]. Errors during surgery can occur due to wrong knee rotation, leading to different ACL bone tunnel orientation [57] or over- or undercorrection during osteotomies [28]. Kawahara et al. [26] analyzed the effect of limb malrotation between 10° ER and 10° IR on preoperative planning of open-wedge HTO. As the HTO opening angle changed only within 0.5° on average, authors concluded that malrotation of the LLR by < 10° does not influence preoperative planning in open-wedge HTO in cases without knee extension deficits. However, extreme care has to be taken in the rotational judgment of LLR in cases with knee extension deficits > 10°, as opening angle differences correlated with knee flexion angles. Knees with > 10° extension deficit had > 1° difference at 10° ER or 10° IR. It has to be noted that limb rotation on LLR was found to be within 36° IR and 22° ER [36, 37]. In patients with osteoarthritis of the knee, 27% of patients who received TKA had a preoperative flexion contracture between 6° and 19° and 9% of patients had severe flexion contracture between 20° and 50° [48]. In addition, radiographs must be interpreted carefully in all circumstances that might cause extension deficits. Postoperative swelling after TKA or HTO and obesity may lead to extension deficits and consequently bias measurements on radiographs. Yoo et al. [64] verified that malrotated limb positioning and flexion were greater on radiographs immediately after TKA surgery than observed at a 1-year follow-up.

The present review has several limitations, mainly emerging from the heterogeneity of the included studies. We did not have access to raw data. Therefore, we were dependent on the given values from the published studies. Additionally, due to the large heterogeneity, a quality assessment of the different studies was not performed. Moreover, we are aware that, besides malrotation, other factors might alter alignment measurements, such as weight-bearing conditions [23, 46, 61] and magnification errors [3]. One general drawback of LLR is that the hindfoot is not included. Thus, the position of the foot cannot be controlled, despite its relationship with lower limb alignment [9, 10, 60]. Weight-bearing LLR for surgical planning of HTO and TKA are still the gold standard assessment for alignment, but other image modalities are available. Patient-specific instrumentation relies on CT scans, which better depict the 3D joint anatomy but not considering the weight-bearing condition [23]. Alternatives that are currently not routinely available in most clinics are weight-bearing CT [5, 17] and the biplanar linear radiograph system (EOS^®^) [12, 20, 50, 58]. EOS^®^ captures anteroposterior and lateral radiographs simultaneously of the whole lower limb during weight bearing [20]. EOS^®^ allows 3D reconstruction, lower radiation dose, and lower measurement error rates due to limb malrotation [11, 12, 20, 41, 58, 59]. Therefore, EOS^®^ has to be considered to replace LLR for surgical planning in the future.

Malalignment leads to the development and progression of osteoarthritis [6, 13, 54], and surgical inaccuracy of deformity correction can lead to inferior clinical outcome and earlier conversion into TKA. The present review shows that malrotation of the limb on preoperative LLR can alter alignment measurements and must be avoided. Radiological assistants, surgeons, and radiologists must be aware of the influence of rotation on alignment measurements, especially in combination with knee flexion. Moreover, further studies analyzing radiographic outcomes of novel patient-specific instrumentation or navigation systems and determining surgical accuracy after HTO [53] have to pay attention to the limb rotation on radiographs. Trials analyzing these developments are often designed to detect small improvements in radiological outcomes or surgical accuracy [25]. Therefore, malrotated LLR can cause a bias in their findings [30].

We recommend the following guidance for daily clinical practice: (1) stitching of the images and the use and position of the magnification device have to be controlled. (2) Attention for accurate limb positioning with the patella straight forward irrespective of the foot position has to be paid. This should be achieved by following the guidelines of Paley et al. [44]. A possible pitfall can occur in subluxated patellae. However, in full knee extension, the patella is usually centered in these cases. Patients with severe distal femoral valgus often have true lateral patellar subluxation in full knee extension. In these cases, the knee extension–flexion axis should be used, and the axis should be positioned parallel to the film. (3) Knee extension deficits should be detected. (4) Before measuring alignment on the conducted LLR, the rotation should be estimated by the patella position or fibular overlap as described above.

## Conclusion

Despite the large heterogeneity between studies, we can conclude that malrotation influences the measured alignment especially when a knee extension deficit is present. Surgeons must be aware when measuring deformities; planning treatments, such as HTO or TKA; and analyzing the surgical outcome. Especially in patients with osteoarthritis with higher prevalence of knee extension deficits or postoperative swelling, the effect of malrotation is significantly higher.

## Appendix

See Table 2.
